# Ultra-Low Core Loss and High-Frequency Permeability Stability in Hot-Press Sintered FeSi Soft Magnetic Composites by Fe_2_O_3_ Nanoparticles Air Gap Filling

**DOI:** 10.3390/ma18092013

**Published:** 2025-04-29

**Authors:** Muhammad Arif, Donghun Han, Wonchan Shin, Seunghun Cha, Changsun Pak, Youngkwang Kim, Sangwoo Kim, Bowha Lee, Jongsoo Rhyee

**Affiliations:** 1 Department of Applied Physics, Institute of Natural Sciences, Kyung Hee University, Yongin 17104, Republic of Korea; muarif786@yahoo.com (M.A.); doontac@khu.ac.kr (D.H.); wkdrns1005@naver.com (W.S.); hun3039@khu.ac.kr (S.C.); cspak1178@gmail.com (C.P.); 2 Technical Research Lab, R-Materials Co., Ltd., Yongin 17111, Republic of Korea; 3 Department of Physics, Oxide Research Center, Hankuk University of Foreign Studies, Yongin 17035, Republic of Koreabwlee@hufs.ac.kr (B.L.)

**Keywords:** soft magnetic composites (SMCs), core loss, FeSi, Fe_2_O_3_ nanopowders, permeability, hot-press

## Abstract

Soft magnetic materials are crucial in motors, generators, transformers, and many electronic devices. We synthesized the FeSi soft magnetic composites (SMCs) with different doping contents of Fe_2_O_3_ nanopowders as fillers via the hot-press sintering technique. This work explores the incorporation of high-resistivity magnetic fillers through a novel compaction technique and investigates the influence of Fe_2_O_3_ nanopowder on the structure and magnetic properties of Fe_2_O_3_ nanopowder-filled composites. The finding reveals that Fe_2_O_3_ nanopowders effectively fill the air gaps between FeSi powders, increasing SMC density. Moreover, all samples exhibit excellent effective permeability frequency stability, ranging from 15 kHz to 100 kHz. Notably, the effective permeability *µ*_e_ improves from 22.32 to 30.45, a 36.42% increase, when the Fe_2_O_3_ doping concentration increases from 0 to 2 wt%. Adding Fe_2_O_3_ nanopowders also enhances electrical resistivity, leading to a 37.21% reduction in eddy current loss in samples for 5 wt% Fe_2_O_3_ addition, compared to undoped samples. Furthermore, as Fe_2_O_3_ content increases from 0 to 5 wt%, the power loss *P*_cv_ of the Fe_2_O_3_-doped Fe-6.5Si SMCs decreases from 25.63 kW/m^3^ to 16.13 kW/m^3^, a 37% reduction. These results suggest that Fe_2_O_3_-doped FeSi SMCs, with their superior soft magnetic properties, hold significant potential for use in high-power and high-frequency electronic applications.

## 1. Introduction

Soft magnetic composites (SMCs) are ferromagnetic materials, synthesized by enclosing them in a thin, resistive insulating layer, forming a core–shell heterogeneous structure [1,2,3,4,5,6]. Because of their distinctive three-dimensional isotropic ferromagnetic behavior, high saturation magnetic flux density, high magnetic permeability, low coercivity, high specific electrical resistivity, and low core loss, SMCs are of great interest to scientists and engineers for use in a variety of electromagnetic devices, including motors, transformers, sensors, and inductors [7,8,9,10]. Among Fe-based SMCs such as Fe-Si, Fe-Si-Al, Fe-Ni, Fe-Ni-Mo, and amorphous SMCs based on their composition [11,12,13,14], the FeSi alloy is preferred for SMCs due to its relatively high resistance and saturation magnetization density [15,16,17]. To meet the growing demands for higher-frequency electronic products, SMCs need high magnetic permeability and low core loss [18,19,20,21,22].

Fe-6.5 wt%Si SMCs have garnered significant attention because of their excellent magnetic properties, including high magnetic permeability, high saturation magnetization, excellent DC bias characteristics, low magnetocrystalline anisotropy, and nearly zero magnetostrictive coefficient [23,24]. However, the brittleness of FeSi results in low compaction density and low permeability [25]. More forming agents and higher compacting pressure are required to achieve desirable properties, although excessive non-magnetic forming agents can reduce the permeability and magnetization of SMCs [15,26]. Excessive pressure during compaction can damage the insulation layer, increasing internal stress and thus reducing permeability and increasing magnetic loss [27]. The advancement of low-loss technologies and the miniaturization of power electronics components depend on optimizing the magnetic properties of SMCs [15]. Therefore, enhancing the high-frequency characteristics of SMCs is essential for specific applications.

To improve the magnetic properties of SMCs, strategies such as particle size matching [28], insulation coating, novel annealing techniques [29], and incorporating tiny, soft metallic magnetic particles [30,31] have been employed. The choice of insulation coating has received significant attention because it has a significant impact on the soft magnetic properties of magnetic powder cores, such as high-frequency loss, frequency stability, and so on. There are two categories of insulation coating: organic and inorganic. Nevertheless, the non-magnetic insulation coating, whether it is organic or inorganic, greatly reduces the saturation magnetization and permeability of magnetic powder cores, which weakens the magnetic properties of magnetic powder cores. Consequently, it is particularly important to use insulation coating with high resistivity and excellent magnetic properties. To address this issue, various ferromagnetic materials such as Mn-Zn ferrites Ni-Zn ferrites, and FexOy have been considered suitable insulators with good soft magnetic properties.

In this context, Liu et al. [1] studied the influence of Fe nanoparticles on the soft magnetic properties of Fe-6.5 wt% Si SMCs. Fe addition enhanced magnetic permeability by up to 24% and maintained comparatively low core loss in Fe-6.5 wt% Si SMCs. Zhao et al. [32] reported FeSi/FeNi SMCs with improved soft magnetic properties for a 22.1% core loss reduction and a 43.8% increase in effective permeability. Wang et al. [33] investigated that Co-doping Fe-6.5Si powder significantly increases electrical resistance and saturation magnetization, with decreased core loss and increased effective permeability. Similarly, Luo et al. [34] studied the effect of Fe_2_O_3_ coating on the structure and magnetic performances of FeSiAl soft magnetic composites prepared via the arc-melting method. The saturation magnetization eventually increased, and coercivity decreased with the increase in Fe_2_O_3_. In addition, the frequency stability for effective permeability was greatly improved. Though most of the research has been done on FeSi using different strategies, core loss in SMCs is still a major concern due to the low electrical resistivity of metallic magnetic nanoparticles and low compaction techniques. Adding high-resistivity magnetic filler (Fe_2_O_3_) is crucial to reducing core loss, particularly eddy current loss, without reducing the magnetic contents of SMC. Additionally, novel compaction techniques like hot-press sintering can further enhance the magnetic properties of SMCs by improving densification, preventing grain growth, and applying axial pressure at high temperatures. This work focuses on the incorporation of high-resistivity magnetic fillers using novel compaction techniques.

Here, we employed oxide magnetic nanoparticles (Fe_2_O_3_) as fillers in FeSi SMCs prepared via the hot-pressing technique and investigated the soft magnetic properties. The appropriate filling of air gaps by Fe_2_O_3_ nanopowders enhances the surface morphology, density, magnetic permeability, and core loss of the SMCs, resulting in ultra-low core loss, which shows promising applicability in various electronic applications.

## 2. Materials and Methods

Commercial gas-atomized FeSi powder (Si content 6.5 wt%, purity surpassing 99.9 wt%) with an average particle size of 30 µm was acquired from Hunan Hualiu New Materials Co., Ltd., Changsha, China. Iron oxide nanoparticles (α-Fe_2_O_3_, 98% purity) with an average particle size of 100 nm were supplied by Sigma Aldrich (St. Louis, MO, USA). Polyvinylpyrrolidone (PVP), Zinc stearate, and ethanol were provided by Sigma Aldrich (Shanghai, China).

**Figure 1:**
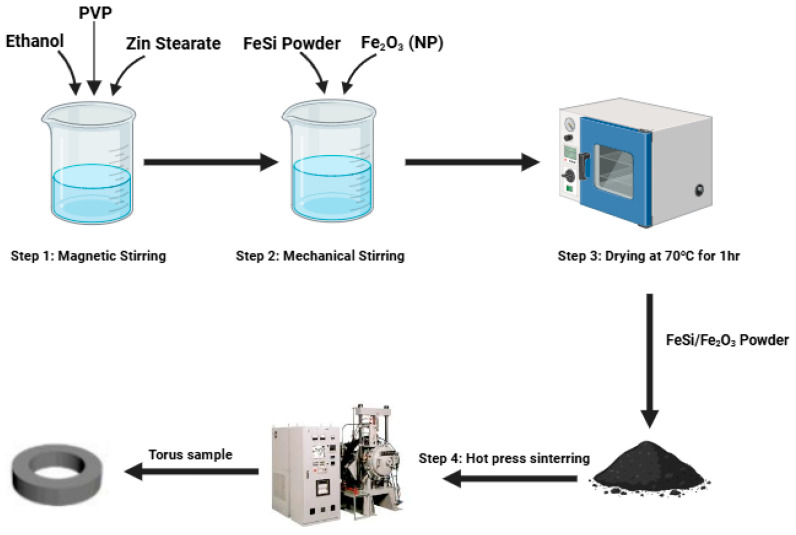
Schematic diagram for FeSi/Fe_2_O_3_ SMCs preparation.

The Fe_2_O_3_ doped Fe-6.5 wt%Si SMCs were made by combining FeSi powders with varying concentrations of Fe_2_O_3_ nanoparticles (0 wt%, 1 wt%, 2 wt%, 3 wt%, 4 wt%, and 5 wt%); 3.0 wt% PVP and 0.5 wt% Zinc stearate were used as binder and lubricant, respectively. The detailed process is given in the schematic diagram shown in Figure 1. Firstly, through magnetic stirring, Zinc stearate and PVP were dissolved completely in ethanol. After adding the FeSi powder and Fe_2_O_3_ nanoparticles, the mixture was mechanically stirred until all the ethanol had been removed. It was then dried for an hour at 70 °C in a vacuum environment. The powdered Fe-6.5 wt%Si-Fe_2_O_3_ composites were then compressed into a toroidal mold with an outside diameter of 20 mm and an inner diameter of 10 mm using a hot-pressing technique under an applied pressure of 70 MPa, after which they were sintered for an hour at 750 °C. Eventually, a systematic characterization of the fabricated Fe_2_O_3_-doped FeSi SMCs was conducted.

The structural properties of the powders were measured using X-ray diffraction (XRD, D8 Advance Bruker, Billerica, MA, USA) with Cu-Kα radiation at a range of 2θ = 10~90°. The surface morphologies and chemical compositions of composites were analyzed using scanning electron microscopy (HR FE-SEM, Gemini360, Carl Zeiss, Zurich, Switzerland) coupled with an energy-dispersive X-ray spectrometer (EDS). The saturation magnetization of the samples was measured by a Physical Property Measurement System (PPMS DynaCool, Quantum Design, San Diego, CA, USA) with an applied maximum field ranging from 0 to 20,000 Oe. To lower the error fraction, the density of the composites was calculated by averaging the sample mass and dimensions. The electrical resistivity was measured using a four-probe system equipped with a Keithly 2400 source meter (Cleveland, OH, USA). For the frequency-dependent effective permeability μ_e_ spectra of the cores, a Precision LCR meter (Key Sight E4980A, Keysight Technologies, Santa Rosa, CA, USA) was used. The *μ*_e_ of the FeSi-Fe_2_O_3_ SMCs was determined from the inductance of the core using the following Equation (1) [35]:

(1) $$\mu_{e}=\frac{Ll_{e}}{\mu_{o}N^{2}A_{e}}$$

where *L* denotes the inductance of the core, *l*_e_ is the effective magnetic circuit length of the toroidal SMCs, *μ*_o_ is the vacuum permeability, *N* is the number of turns of insulated copper wire twists on the core, and *A*_e_ is the SMCs effective cross-section area. To explore the effect of nanopowders on the losses, the frequency-dependent core loss measurement was carried out via an AC loop tracer (IWATSU B-H ANALYZER, SY-8219, Iwatsu Corporation, Tokyo, Japan).

## 3. Results

**Figure 2:**
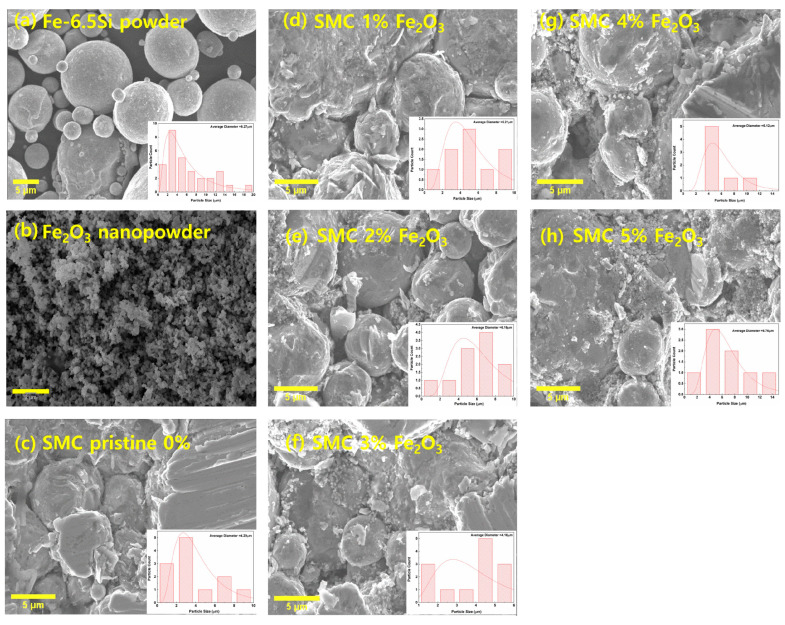
SEM images of (**a**) Raw FeSi powder, (**b**) Fe_2_O_3_ nanopowders, Fe-6.5 wt%Si-Fe_2_O_3_ SMCs with (**c**) 0 wt%, (**d**) 1 wt%, (**e**) 2 wt%, (**f**) 3 wt%, (**g**) 4 wt%, and (**h**) 5 wt% of Fe_2_O_3_ nanoparticles. The inset shows the particle size distribution for FeSi SMCs.

Figure 2a, b, and c–h display the SEM images for raw FeSi, Fe_2_O_3_ nanopowders, and SMCs, with an inset graph showing particle size distribution for FeSi, respectively. The SEM image in Figure 2a shows that FeSi particles are spherical, and the surface is quite smooth. The average particle size is about 10~30 μm. The Fe_2_O_3_ nanopowders are aggregated, in addition to a uniformly smooth spherical shape, as shown in Figure 2b. The SEM images for all prepared SMCs with different Fe_2_O_3_ content are illustrated in Figure 2c–h, which shows that adding NP from 0 to 2 wt% filled the pores, indicating increased compact density. However, the excessive addition of NP gives rise to more pores due to aggregation, resulting in a reduced density. The SMC of Fe_2_O_3_ content 2 wt% becomes a compact distribution and a well-connected network between grains.

**Figure 3:**
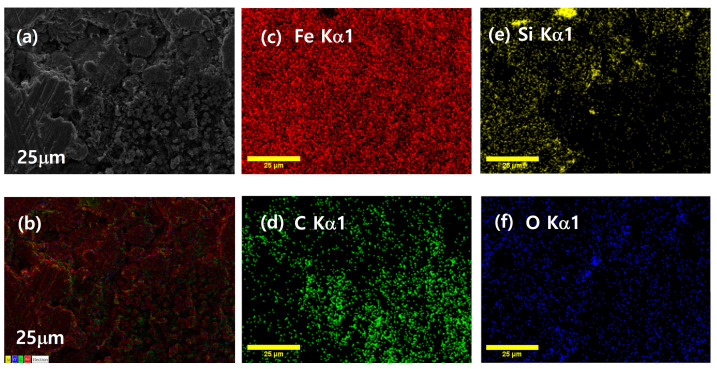
(**a**,**b**) SEM image of polished surface (**c**–**f**) EDS maps of corresponding FeSi-Fe_2_O_3_ (2 wt%) SMCs.

Figure 3a,b and c–f depict the SEM images and the corresponding EDS elemental distribution maps for the FeSi-Fe_2_O_3_ SMCs, respectively. The result demonstrates that there is a uniform distribution of Fe both inside and between the particle regions. This behavior validates the dispersion of the Fe nanoparticles in the pores between the micron-sized FeSi particles. On the other hand, the Si is observed in the particle region only; however, O and C elements are primarily found in the space between the particles.

**Figure 4:**
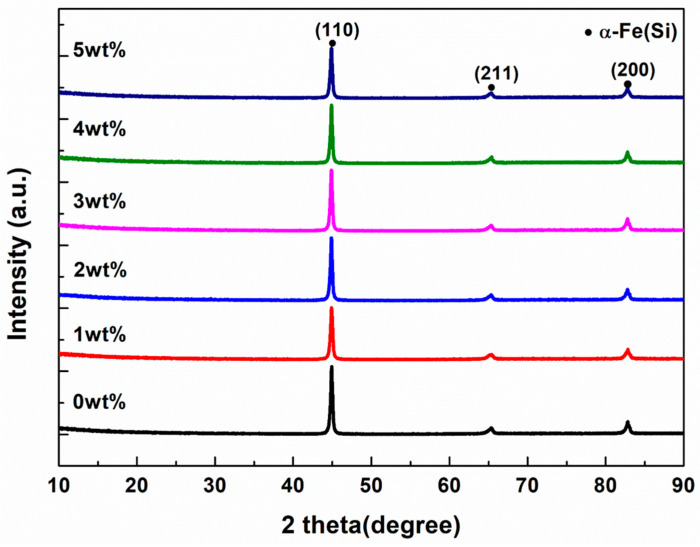
XRD of FeSi-Fe_2_O_3_ (0, 1, 2, 3, 4, and 5 wt%) SMCs.

The XRD patterns of FeSi-Fe_2_O_3_ SMCs with varying concentrations of Fe_2_O_3_ NP are presented in Figure 4. In all samples, three distinct characteristic peaks are observed at 44.7°, 65.1°, and 82.5° that correspond to the (110), (200), and (211) bcc crystal structure of the α-Fe (Si) phase. Furthermore, there is no obvious change in the intensity or peak shift when adding Fe_2_O_3_ nanopowders.

**Figure 5:**
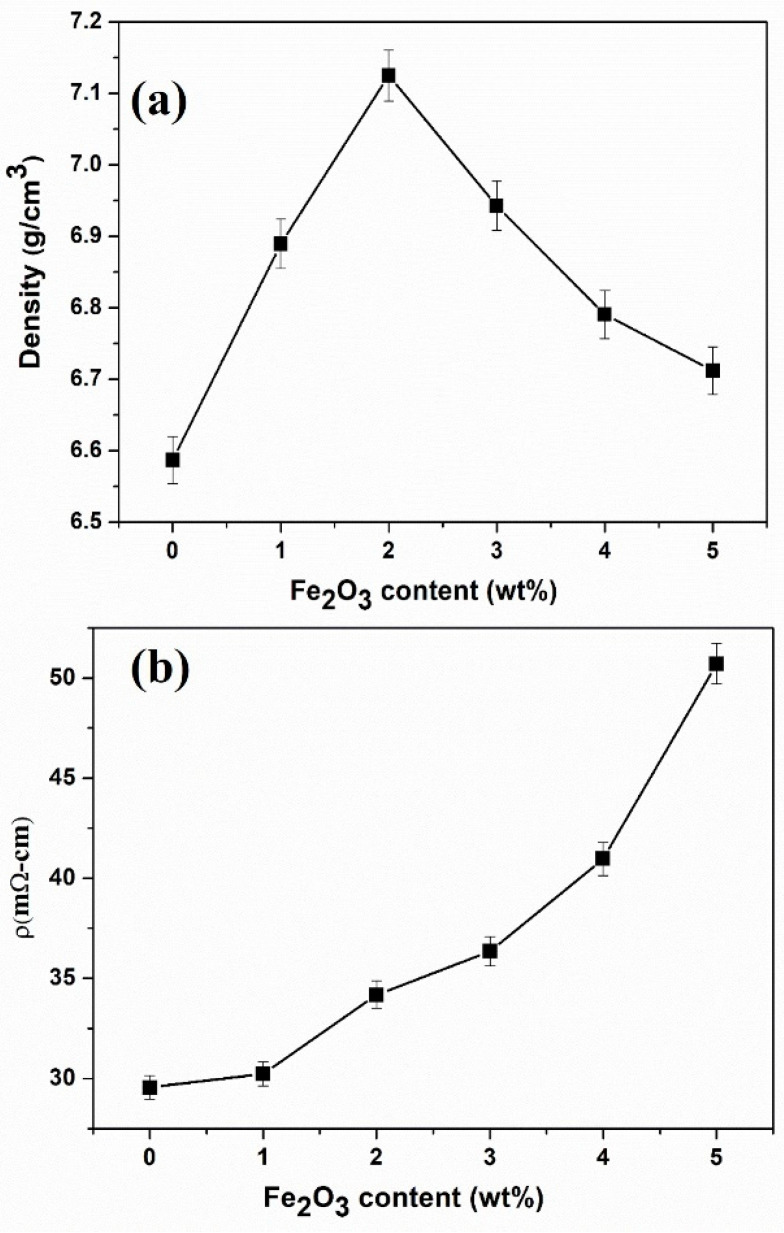
(**a**) Density and (**b**) electrical resistivity of FeSi-Fe_2_O_3_ SMC samples, respectively.

The density and electrical resistivity of FeSi-Fe_2_O_3_ SMCs are presented in Figure 5a,b, respectively. The density of FeSi-Fe_2_O_3_ SMCs first increased, reaching a maximum value (7.12 g/cm^3^) for a sample with 2 wt% of Fe_2_O_3_, which is 8.2% higher than 6.58 g/cm^3^ for the sample with the pristine one (0 wt% of Fe_2_O_3_), and then decreased with a further increase in Fe_2_O_3_ nanoparticle contents. This increase supports the assumption that the nanoparticles effectively fill interparticle voids between FeSi particles, leading to improved compaction and reduced porosity. With a further increase in Fe_2_O_3_ nanoparticles, additional pores are created due to agglomeration, which lowers the density of SMCs. Conversely, the electrical resistivity (shown in Figure 5b) gradually increased from 29.55 to 50.70 mΩ-cm with increasing contents of Fe_2_O_3_ NP from 0–5 wt%, respectively. Interestingly, the sample with the high doping concentration of Fe_2_O_3_ (5 wt%) has the highest value (50.70 mΩ-cm), which is 71.57% higher than the pristine sample 0 wt% of Fe_2_O_3_ (29.55 mΩ-cm). These results are reasonable because doping with an insulating material can cause high electrical resistivity. Notably, the electrical resistivity values for our FeSi-Fe_2_O_3_ SMCs are much higher than those reported [32].

**Figure 6:**
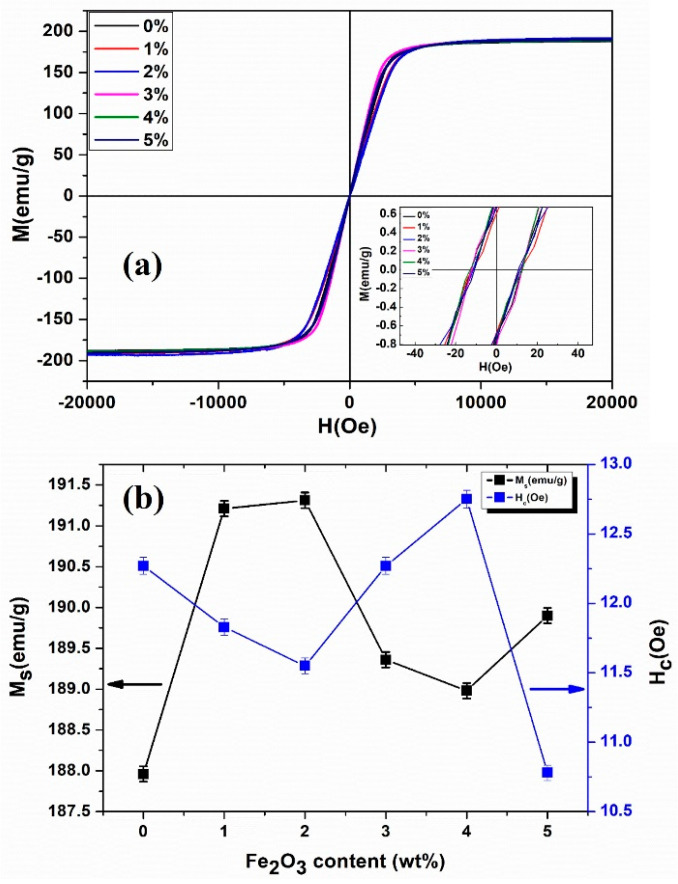
(**a**) MH hysteresis loops of FeSi-Fe_2_O_3_ SMCs, with an Inset graph to see the H_c_ (**b**) M_s_ (left axis) and H_c_ (right axis) with Fe_2_O_3_ contents.

The room temperature magnetic hysteresis loop measurement (MH loops) for all samples with different doping concentrations of Fe_2_O_3_ are shown in Figure 6a. Notably, all the coated samples exhibited good soft magnetic performance with high M_s_ and very low H_c,_ as presented in Table 1. Inset of Figure 6a is the expanded plot near the low field range (|H| < 40 Oe) to see the H_c_ more clearly. To further evaluate the dependence of M_s_ and H_c_ on doping concentration, the extracted parameters from MH loops are plotted against Fe_2_O_3_ contents, as illustrated in Figure 6b. Interestingly, the value of saturation magnetization increases first from 187.96 to 191.31 emu/g as the amount of Fe_2_O_3_ increases from 0% to 2%, then decreases to 189.90 emu/g with the further increase in Fe_2_O_3_ content from 2 to 4 wt%, and then increases again in the last sample with 5 wt% of Fe_2_O_3_ nanopowders. This small enhancement in M_s_ can be attributed to the low saturation magnetization of Fe_2_O_3_ (0.726 emu/g). Introducing Fe_2_O_3_ NP raises the ferromagnetic filling factor by filling the pores in the SMCs and increasing the saturation magnetization. However, doping an excess amount of Fe_2_O_3_ induces new pores due to agglomeration, which in turn decreases the volume fraction of the ferromagnetic phase, resulting in a decrease in M_s_ [36]. However, it increased again in the last sample with 5 wt% content of Fe_2_O_3_. In contrast, the value of Hc followed an opposite trend to M_s_. The H_c_ value drops first from 12.27 to 11.55 Oe with increased Fe_2_O_3_ contents from 0 to 2 wt% and then follows an increasing trend up to 12.75 Oe with a further increase in Fe_2_O_3_ nanopowders. Moreover, the lowest value for a sample with 5 wt% doping content of Fe_2_O_3_ is observed around 10.78 Oe. The results are well understood because it is well-known that the coercive field strongly depends on the crystal structure and defects such as porosity, secondary phases, and interface [37]. Interestingly, the values of H_c_ for all our samples are much lower in comparison to the reported results [1].

**Figure 7:**
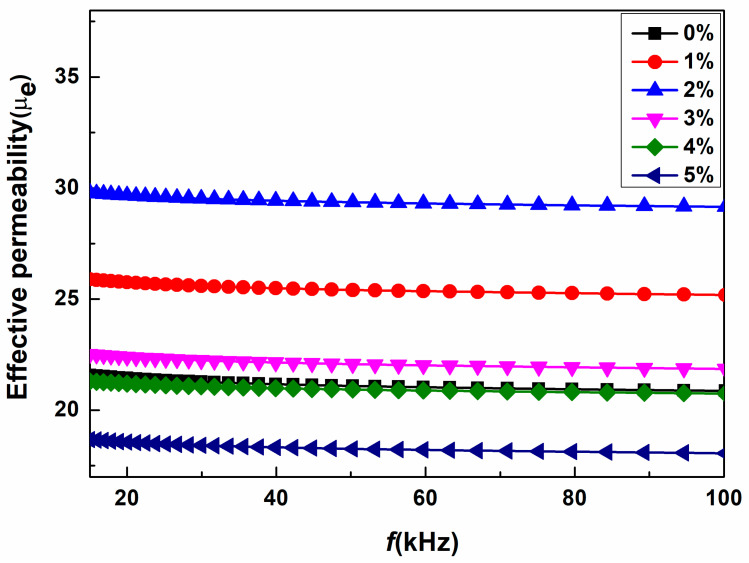
Frequency-dependent effective permeability spectra for all FeSi-Fe_2_O_3_ samples.

Figure 7 illustrates the frequency-dependent effective permeability μ_e_ of SMCs with varying content of Fe_2_O_3_ in the range of 15 kHz to 100 kHz. Due to the growing demand for high-frequency applications of electronic devices, the effective permeability of SMCs must generally remain constant up to high frequencies. Interestingly, all our compact samples showed excellent frequency stability of effective permeability ranging from 10 kHz to 100 kHz. Furthermore, as the Fe_2_O_3_ content increased from 0 to 2 wt%, the effective permeability μe increased from 22.32 to 30.45. However, as the Fe_2_O_3_ quantities increased further, the value of μe decreased to 19.32. The maximum value observed for the 2 wt% of NP content is 36.42 %, which is higher than the sample without doping Fe_2_O_3_. For SMC, the effective permeability can be expressed as [38]:

(2) $$\mu_{e}=\frac{3+\left(\mu^{\prime }-1\right)\left(3-3g\right)}{3+g\left(\mu^{\prime }-1\right)}$$

where μ′ indicates the permeability of the magnetic powders and g is the volume fraction of non-magnetic substances. The effective permeability is correlated with the density and saturation magnetization of the SMCs, as per the equation above. The effective permeability of the magnetic powders is proportional to the density and the square of the M_s_ [39,40,41]. The incorporation of Fe_2_O_3_ nanopowders from 0 to 2 wt% enhances the density, saturation magnetization (M_s_), and relative permeability. Adding Fe_2_O_3_ nanopowders beyond 2 wt% decreases both M_s_ and density, thereby reducing the effective permeability.

**Figure 8:**
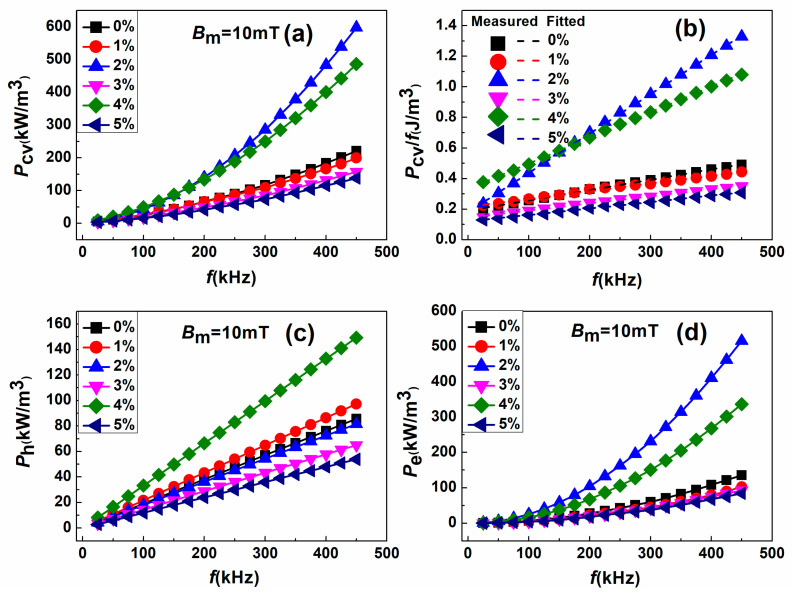
(**a**) Total core loss P_cv_, (**b**) P_cv_/f, (**c**) hysteresis loss P_h_, and (**d**) eddy current loss P_e_ as a function of frequency for FeSi-Fe_2_O_3_ SMC samples.

To assess the impact of magnetic nanopowders on the AC magnetic property of SMCs, the total core loss (P_cv_) versus frequency (f) plots for all Fe_2_O_3_-doped Fe-6.5wt%Si SMCs measured at an applied magnetic field of 10 mT in the frequency range of 15 to 100 kHz are shown in Figure 8a. The P_cv_ of Fe_2_O_3_-doped Fe-6.5wt%Si SMC progressively drops from 25.63 kW/m^3^ to 16.13 kW/m^3^ as Fe_2_O_3_ contents rise from 0 wt% to 5 wt%, as presented in Table 2. Interestingly, for the sample with maximum Fe_2_O_3_ content (5 wt%), the P_cv_ is reduced by 35% compared to undoped FeSi (0 wt% of Fe_2_O_3_) (P_cv_ = 16.13 kW/m^3^, f =100 kHz, and B_m_ = 10 mT). Moreover, the P_cv_ for our compacted samples is much lower than those of previous results on FeSi SMCs [42,43,44]. This is mostly because of the high resistivity of Fe_2_O_3_, which blocks the interaction between magnetic particles and lowers the total loss.

According to Bertotti’s classical theory of loss separation, P_cv_ primarily falls into three categories: hysteresis losses (Ph), eddy current losses (Pe), and residual losses (Pr), as follows [45]:

(3) $$P_{cv}=P_{e}+P_{h}+P_{r}$$

where P_cv_ represents the total core loss, P_h_ is the hysteresis loss, P_e_ is the eddy current loss, and P_r_ is the residual or excessive loss. In power applications, P_r_ often has a very low value in comparison to P_h_ and P_e_ [46]. Consequently, in our situation, it may be disregarded, and the simplified equation is as follows:

(4) $$P_{cv}=P_{e}+P_{h}$$

Hysteresis loss signifies the energy lost per unit volume during a single magnetization cycle, and it can be written as follows:

(5) $$P_{h}=f\emptyset HdB=k_{h}B_{m}^{3}f$$

where B_m_ represents the applied magnetic flux density, f is the magnetic field frequency, and k_h_ is the hysteresis loss coefficient.

The eddy current loss that results from the application of Faraday’s law to ferromagnetic materials magnetized in an alternating magnetic field can be described by the following equation [2,47]:

(6) $$P_{e}=\frac{k_{e}B_{m}^{2}d_{eff}^{2}}{\rho }f^{2}$$

where k_e_ is the eddy current loss coefficient, d_eff_ is the effective diameter of the particles, B_m_ is the applied magnetic flux density, and ρ is the electrical resistivity of SMCs. Combining Equations (3) and (4), the above Equation (2) can be rewritten as:

(7) $$P_{cv}=k_{h}\times f+k_{e}\times f^{2}$$

Hysteresis loss and eddy current loss are known to be proportional to frequency (f) and the square of frequency (f^2^), respectively. Kollar’s loss separation model states that the total energy loss W_t_ (J/m^3^) is determined by dividing Equation (5) by the frequency as given below:

(8) $$W_{t}=\frac{P_{cv}}{f}=k_{h}+k_{e}\times f$$

The P_cv_/f versus f fitted curves with linear Equation (5) for all the SMCs with different Fe_2_O_3_ contents are shown in Figure 8b. Our experimental results (solid dots) agreed well with the corresponding fits (dashed lines). The k_h_ and k_e_ are derived from the intercept and slope of the fits, respectively [37]. Using the fitting formula, the hysteresis losses Ph and eddy current loss Pe are extracted and plotted against f, as seen in Figure 8c and Figure 8d, respectively.

The value of P_h_ at f = 100 kHz first dropped from 19.022 kW/m^3^ to 14.43 as the Fe_2_O_3_ contents changed from 0 to 3wt%, as shown in Figure 8c. It subsequently increased to 33.19 kW/m^3^ for the sample with 4 wt% Fe_2_O_3_ contents and then dropped again to 11.97 kW/m^3^ for the sample with 5 wt% Fe_2_O_3_. It is commonly known that hysteresis loss and coercivity are directly related [1]. Therefore, the changing trend of H_c_, which coincides with the altering trend of P_h_, may be one possible explanation. In our case, the lowest P_h_ value recorded for a sample containing 5 wt% of Fe_2_O_3_ is approximately 11.97 kW/m^3^.

**Figure 9:**
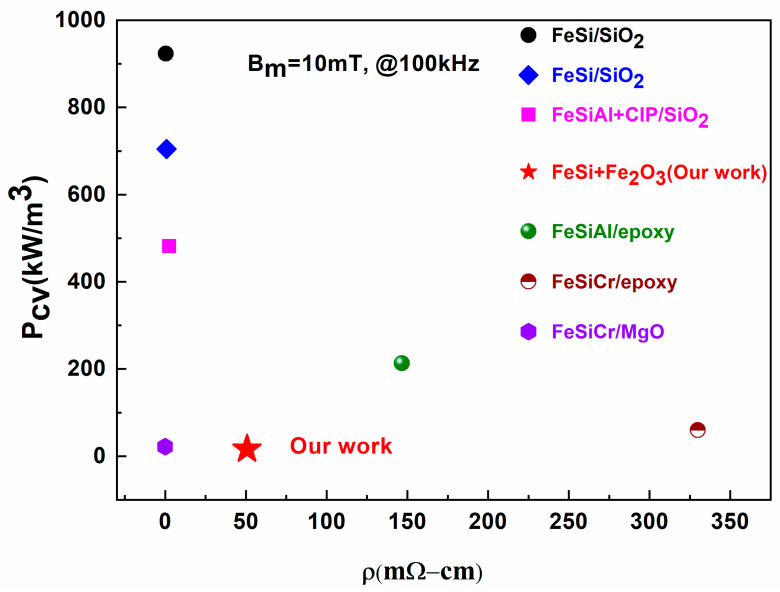
Performance comparison of FeSi-Fe_2_O_3_ SMCs with the previously reported Fe-based SMCs [40,41,42,46,47,48,49,50].

In contrast, the P_e_ versus f plot shown in Figure 8d followed a gradually decreasing trend with the increase in Fe_2_O_3_ contents, except for the sample with 2 wt% and 4 wt% of Fe_2_O_3_ contents. The value of P_e_ at f = 100 kHz declined from 6.61 to 4.15 kW/cm^3^ as the Fe_2_O_3_ contents changed from 0 to 5 wt%. Since Pe is inversely proportional to electrical resistivity according to Equation (6), the increased electrical resistivity of SMCs might be the possible reason for the decrease in P_e_, as indicated in Table 2. Consequently, the SMC particles’ surface can be coated with a high electrical resistive insulating layer to efficiently inhibit eddy currents between them, thereby lowering the P_e_. For the samples with 2 wt% and 4 wt% of Fe_2_O_3_, P_e_ is around 25.33 kW/m^3^ and 16.07 kW/m^3^, respectively. This can be ascribed to higher density because when magnetic particles (e.g., FeSi) are closely packed or poorly insulated, electrical paths can form between them, allowing eddy currents to circulate more easily under alternating magnetic fields. This, in turn, increases Pe. Interestingly, the P_e_ is greatly decreased by 37.21% when 5 wt% Fe_2_O_3_ NP is added. Figure 9 displays the comparison of the high-frequency magnetic performance of FeSi/Fe_2_O_3_(NP) SMCs in this work with previously reported different Fe-based SMCs. The remarkable soft magnetic properties of FeSi-Fe_2_O_3_ SMCs in this work will find widespread use in high-power and high-frequency electronic applications. Further lowering eddy current losses in the high-frequency range and methodically examining the composites’ mechanical and thermal properties over extended operating conditions are the goals of our upcoming studies.

## 4. Conclusions

In summary, the soft magnetic characteristics of Fe-6.5wt%Si-Fe_2_O_3_ SMCs prepared via the hot-press technique with different doping concentrations of Fe_2_O_3_ (0–5 wt%) were systematically studied. The incorporation of high-resistivity Fe_2_O_3_ effectively filled interparticle gaps, increased density, and enhanced resistivity. As a result, the effective permeability significantly improved from 22.32 to 30.45 (a 36.4% increase), while eddy current loss decreased by 37.2% at 5 wt% Fe_2_O_3_. Furthermore, increasing Fe_2_O_3_ content also decreased the total core loss from 25.63 to 16.13 kW/m^3^. These results show that FeSi/FeO_3_ SMCs have a great deal of potential for high-frequency power applications.

## Figures and Tables

**Table 1:** M_s_ and H_c_ of FeSi-Fe_2_O_3_ SMCs with different Fe_2_O_3_ nanoparticle contents.

Contents wt%	0 wt%	1 wt%	2 wt%	3 wt%	4 wt%	5 wt%
M_s_	187.6	191.21	191.31	189.36	188.98	189.9
H_c_	12.27	11.83	11.55	12.27	12.75	10.78

**Table 2:** Variations in permeability, the core loss, coercivity, density, and electrical resistivity of SMCs versus Fe_2_O_3_ nanoparticle contents.

Contents wt%	P_cv_(kW/m^3^) f = 100 kHz, B_m_ = 10 mT	P_h_(kW/m^3^) f = 100 kHz, B_m_ = 10 mT	P_e_(kW/m^3^) f = 100 kHz, B_m_ = 10 mT	μ_e_	ρ (g/cm^3^)	ρ (mΩ-cm)
0	25.63	19.02	6.61	21.57	6.56	29.55
1	26.68	21.61	5.07	25.89	6.88	30.23
2	43.45	18.11	25.33	30.05	7.12	34.18
3	18.05	14.43	4.38	22.51	6.94	36.34
4	49.26	33.19	16.07	21.29	6.79	40.96
5	16.13	11.97	4.153	18.67	6.71	50.70

## Data Availability

The original contributions presented in this study are included in the article. Further inquiries can be directed to the corresponding author.

## References

[1] Liu D., Liu X., Wang J., Mao X., Xu X. (2020). The influence of Fe nanoparticles on microstructure and magnetic properties of Fe-6.5 wt% Si soft magnetic composites. J. Alloys Compd..

[2] Perigo E.A., Weidenfeller B., Kollár P., Füzer J. (2018). Past, present, and future of soft magnetic composites. Appl. Phys. Rev..

[3] Peng Y., Nie J., Zhang W., Ma J., Bao C., Cao Y. (2016). Effect of the addition of Al_2_O_3_ nanoparticles on the magnetic properties of Fe soft magnetic composites. J. Magn. Magn. Mater..

[4] Wu S., Sun A., Zhai F., Wang J., Zhang Q., Xu W., Logan P., Volinsky A.A. (2012). Annealing effects on magnetic properties of silicone-coated iron-based soft magnetic composites. J. Magn. Magn. Mater..

[5] He W., Li H., Han X., Wang X., Wang G., Zhang X., Shcheretskyi O. (2024). High-temperature dry sliding friction and wear behavior of in-situ (Al_3_Zr + ZrB_2_)/AA6016 aluminum matrix composites. Mater. Today Commun..

[6] Wang G., Li H., Jiao L., Zhang X., Wang X., Shen W., Zhang C. (2024). Effect of rotational speed on friction stir welding microstructure and properties of cast and rolled (ZrB_2_ + Al_3_Zr) particle-reinforced aluminum matrix composites. J. Mater. Sci..

[7] Wu Y., Bitoh T., Hono K., Makino A., Inoue A. (2001). Microstructure and properties of nanocrystalline Fe–Zr–Nb–B soft magnetic alloys with low magnetostriction. Acta Mater..

[8] Kim Y.B., Jang D., Seok H., Kim K. (2007). Fabrication of Fe–Si–B based amorphous powder cores by cold pressing and their magnetic properties. Mater. Sci. Eng. A.

[9] Schoppa A., Delarbre P. (2014). Soft magnetic powder composites and potential applications in modern electric machines and devices. IEEE Trans. Magn..

[10] Gilbert I., Moorthy V., Bull S., Evans J., Jack A. (2002). Development of soft magnetic composites for low-loss applications. J. Magn. Magn. Mater..

[11] Sundar R., Deevi S. (2005). Soft magnetic FeCo alloys: Alloy development, processing, and properties. Int. Mater. Rev..

[12] Streckova M., Medvecky L., Füzer J., Kollár P., Bures R., Faberova M. (2013). Design of novel soft magnetic composites based on Fe/resin modified with silica. Mater. Lett..

[13] Schäfter T., Burghaus J., Pieper W., Petzoldt F., Busse M. (2015). New concept of Si–Fe based sintered soft magnetic composite. Powder Metall..

[14] Krings A., Boglietti A., Cavagnino A., Sprague S. (2016). Soft magnetic material status and trends in electric machines. IEEE Trans. Ind. Electron..

[15] Zheng Z., Li S., Peng K. (2023). Magnetic properties regulation and loss contribution analysis of FeSi soft magnetic composites doped by carbonyl iron powders. J. Magn. Magn. Mater..

[16] Luo Z., Hu W., Luo F., Li Y., Wang J., Liu X. (2019). Enhanced magnetic properties and reduced core loss of intergranular insulating Fe-Si soft magnetic composites with three-shell SiO_2_-Fe_2_SiO_4_-SiO_2_ insulating layer. J. Solid State Chem..

[17] Fan X., Wu Z., Li G., Wang J., Xiang Z., Gan Z. (2016). High resistivity and low core loss of intergranular insulated Fe–6.5 wt.% Si/SiO_2_ composite compacts. Mater. Des..

[18] Zhao Y.W., Zhang X., Xiao J.Q. (2005). Submicrometer Laminated Fe/SiO_2_ Soft Magnetic Composites—An Effective Route to Materials for High-Frequency Applications. Adv. Mater..

[19] Frayman L., Quinn S., Quinn R., Green D., Hanejko F. (2015). Advanced soft magnetic composite materials for AC applications with reduced iron losses. Powder Metall..

[20] Wang M., Zan Z., Deng N., Zhao Z. (2014). Preparation of pure iron/Ni–Zn ferrite high strength soft magnetic composite by spark plasma sintering. J. Magn. Magn. Mater..

[21] Páez-Pavón A., Jiménez-Morales A., Santos T., Quintino L., Torralba J. (2016). Influence of thermal debinding on the final properties of Fe–Si soft magnetic alloys for metal injection molding (MIM). J. Magn. Magn. Mater..

[22] Zhou T., Liu Y., Cao P., Du J., Lin Z., Wang R., Jin L., Lian L., Harris V.G. (2020). Cold Sintered Metal–Ceramic Nanocomposites for High-Frequency Inductors. Adv. Electron. Mater..

[23] Fu H., Mo Y., Zhang Z., Xie J. (2016). Ultra-low deformation rate sensitivity of columnar-grained Fe-6.5 wt% Si alloy with <100> orientation. Mater. Sci. Eng. A.

[24] Fu H., Zhang Z., Jiang Y., Xie J. (2016). Applying the grain orientation dependence of deformation twinning to improve the deformation properties of an Fe-6.5 wt% Si alloy. J. Alloys Compd..

[25] Li W., Xiao S., Li W., Ying Y., Yu J., Zheng J., Qiao L., Li J., Naoki W., Wu J. (2022). Hybrid amorphous soft magnetic composites with ultrafine FeSiBCr and submicron FeBP particles for MHz frequency power applications. J. Magn. Magn. Mater..

[26] Liu M., Huang K., Liu L., Li T., Cai P., Dong Y., Wang X.-M. (2018). Fabrication and magnetic properties of novel Fe-based amorphous powder and corresponding powder cores. J. Mater. Sci. Mater. Electron..

[27] Yu H., Li J., Li J., Chen X., Han G., Yang J., Chen R. (2022). Enhancing the properties of FeSiBCr amorphous soft magnetic composites by annealing treatments. Metals.

[28] Haibo S., Ce W., Changbao Z., Jinghui W. (2022). High-frequency loss analysis and related magnetic properties of Fe-based amorphous soft magnetic composites with different granularity matches. J. Appl. Phys..

[29] Li Z., Dong Y., Pauly S., Chang C., Wei R., Li F., Wang X.-M. (2017). Enhanced soft magnetic properties of Fe-based amorphous powder cores by longitude magnetic field annealing. J. Alloys Compd..

[30] Wang J., Guo Z., Zeng Q., Hang G., Xue Z., Chen D., Liang Z., Sun H. (2020). Magnetic properties regulation and loss contribution analysis for Fe-based amorphous powder cores doped with micron-sized FeSi powders. J. Magn. Magn. Mater..

[31] Shi G., Li M., Wang H., Yin G., Wang M., Wang L. (2024). Effect of the shape and content for added iron powder on properties of nanocrystalline soft magnetic composites. Mater. Today Commun..

[32] Zhao R., Huang J., Yang Y., Jiao L., Dong Y., Liu X., Liu Z., Wu S., Li X., He A. (2022). The influence of FeNi nanoparticles on the microstructures and soft magnetic properties of FeSi soft magnetic composites. Adv. Powder Technol..

[33] Wang J., Liu X., Lei C., Mao X., Liu D., Luo Z., Luo F. (2020). Core loss reduction for Fe-6.5 wt% Si soft magnetic composites doped with Co element. J. Magn. Magn. Mater..

[34] Luo F., Luo Z., Hu W., Wang J., Wu Z., Li G., Li Y., Liu X. (2019). Influences of Fe_2_O_3_ content on structure and magnetic performances of FeSiAl soft magnetic composites. Mater. Res. Express.

[35] Liu J., Dong Y., Zhu Z., Zhao H., Pang J., Wang P., Zhang J. (2022). Fe-based amorphous magnetic powder cores with low core loss fabricated by novel gas–water combined atomization powders. Materials.

[36] Zhang C., Zhang W., Yuan W., Peng K. (2021). Preparation and magnetic properties of core–shell structured Fe-Si/Fe3O4 composites via in-situ reaction method. J. Magn. Magn. Mater..

[37] Liu X., Zhang Z., Guo W., Zhang R., Xu F. (2024). Ultra-low core loss and high permeability Fe-based amorphous soft magnetic composites with ultra-fine FeNi additives. J. Mater. Sci. Mater. Electron..

[38] Anhalt M., Weidenfeller B. (2009). Theoretical and experimental approach to characteristic magnetic measurement data of polymer bonded soft magnetic composites. J. Appl. Phys..

[39] Liu Z., Dong Y., Liu X., Lu H., Wu Y., Zhang H., He A., Li J., Wang X. (2021). Microstructure and soft magnetic properties of Fe_85−x_Si_9.6_Al_5.4_Ti_x_ composite magnetic powder cores. J. Alloys Compd..

[40] Abdel-Aal S.K., Abdel-Rahman A.S. (2019). Fascinating physical properties of 2D hybrid perovskite [(NH_3_)(CH_2_)_7_(NH_3_)]CuCl_x_Br_4−x_, x = 0, 2 and 4. J. Electron. Mater..

[41] Wu T., Ju D., Wang C., Huang H., Li C., Wu C., Wang C., Liu H., Jiang X., Ye K. (2023). Ferrite materials with high saturation magnetic induction intensity and high permeability for magnetic field energy harvesting: Magnetization mechanism and Brillouin function temperature characteristics. J. Alloys Compd..

[42] Qiu Y., Wang R., He Y., Kong H., Li S., Wu Z. (2022). Effects of axial pressure on the evolution of core–shell heterogeneous structures and magnetic properties of Fe–Si soft magnetic powder cores during hot-press sintering. RSC Adv..

[43] Wang R., He Y., Kong H., Wang J., Wu Z., Wang H. (2022). Influence of sintering temperature on heterogeneous-interface structural evolution and magnetic properties of Fe–Si soft magnetic powder cores. Ceram. Int..

[44] Wang R., Huang H., Li K., Yang J., Wu Z., Kong H. (2024). Design and evolution of Fe–Si–Al soft magnetic composites doped with carbonyl iron powders: Overcoming the restrictive relation between permeability and core loss. Ceram. Int..

[45] Bertotti G. (1988). General properties of power losses in soft ferromagnetic materials. IEEE Trans. Magn..

[46] Zhang Y., Huijuan J., Ying S. (2000). General properties of low-frequency power losses in Fe-based nanocrystalline soft magnetic alloys. J. Mater. Sci. Technol..

[47] Füzerová J., Füzer J., Kollár P., Bureš R., Fáberová M. (2013). Complex permeability and core loss of soft magnetic Fe-based nanocrystalline powder cores. J. Magn. Magn. Mater..

[48] Peng X., Zhang A., Li J., Yu S., Chang J., Ge M., Yang Y., Xu J., Hong B., Jin D. (2019). Design and fabrication of Fe–Si–Al soft magnetic composites by controlling orientation of particles in a magnetic field: Anisotropy of structures, electrical and magnetic properties. J. Mater. Sci..

[49] Choi Y.J., Ahn J.H., Kim D.H., Kim Y.R., Lee B.W. (2022). Core-loss reduction of Fe–Si–Cr crystalline alloy according to particle size in the high frequency band. Curr. Appl. Phys..

[50] Wu S., Dong Y., Li X., Gong M., Zhao R., Gao W., Wu H., He A., Li J., Wang X. (2022). Microstructure and magnetic properties of FeSiCr soft magnetic powder cores with a MgO insulating layer prepared by the sol-gel method. Ceram. Int..

